# Treatment of Methicillin-Resistant *Staphylococcus aureus* (MRSA) Bacteremia—Guidelines and Recent Developments

**DOI:** 10.3390/medicines13020018

**Published:** 2026-05-30

**Authors:** Ashlesha Kaushik, Julia Fomicheva, Kimberly Zellmer, Corey Thieman, Sandeep Gupta

**Affiliations:** 1 Division of Pediatric Infectious Diseases, Unity Point Health at St. Luke’s Regional Medical Center, University of Iowa Carver College of Medicine, 2720 Stone Park Blvd, Sioux City, IA 51104, USA; 2 Cambridge Health Alliance, Division of Primary Care Behavioral Health Integration, Harvard Medical School, 25 Shattuck St., Boston, MA 02115, USA; julia_fomicheva@hms.harvard.edu; 3 Division of Pharmacology, Unity Point Health at St. Luke’s Regional Medical Center, 2720 Stone Park Blvd, Sioux City, IA 51104, USA; kimberly.zellmer@unitypoint.org (K.Z.); corey.thieman@unitypoint.org (C.T.); 4 Division of Pulmonary and Critical Care, Unity Point Health at St. Luke’s Regional Medical Center, 2720 Stone Park Blvd, Sioux City, IA 51104, USA; sandeep.gupta@unitypoint.org

**Keywords:** methicillin-resistant *Staphylococcus aureus*, MRSA, bacteremia, treatment, antimicrobials

## Abstract

Methicillin-resistant *Staphylococcus aureus* (MRSA) remains a major cause of serious infection and is associated with substantial morbidity and mortality. Clinical presentations range from localized disease to severe, life-threatening infections, including bacteremia and sepsis with metastatic complications such as infective endocarditis and osteoarticular involvement. MRSA bacteremia carries a high risk of dissemination and death, underscoring the importance of early recognition and effective management. Optimal treatment requires timely initiation of appropriate antimicrobial therapy in conjunction with source control when indicated. Despite advancements in treatment, persistent MRSA bacteremia continues to pose significant clinical challenges. Given the complexity of these infections, a clear understanding of current treatment strategies is essential for clinicians. In this narrative review, we summarize contemporary guideline-based approaches to the management of MRSA bacteremia, highlight key pharmacologic considerations of available antimicrobial agents, and discuss knowledge gaps and recent developments in both established and emerging therapies aimed at improving outcomes in these challenging infections.

## 1. Introduction

MRSA, first observed in the 1960s [1], can cause varying disease presentations, ranging from skin and soft tissue infections to bloodstream infections [2,3]. MRSA has evolved to develop several mechanisms to evade host immunity, manifest antimicrobial-resistance, and form biofilms that enable MRSA to cause persistent and recurrent infections. Methicillin-resistant *Staphylococcus aureus* (MRSA) is a frequent cause of *Staphylococcus aureus*-associated bacteremia (SAB), with reported incidence of SAB ranging between 9.3 and 65 per 100,000 person-years [4]. MRSA bacteremia can often be persistent, leading to serious manifestations, such as endocarditis, meningitis, and bone and joint infections, and it continues to be associated with high mortality rates, underlining the need for prompt recognition and appropriate treatment [1,2,5]. Timely management is crucial and involves aggressive source control, as well as appropriate use of MRSA antimicrobials. Even with advances in treatment, significant gaps and controversies remain in management of persistent MRSA bacteremia. In this narrative review, we aim to discuss the epidemiology and predisposing factors for *S. aureus* bacteremia, with a focus on MRSA, and describe the current State-of-the-Art treatment guidelines for MRSA bacteremia, discuss emerging antimicrobial regimens and adjunctive therapies, and address critical knowledge gaps and current practices in the management of persistent MRSA bacteremia. For this narrative review, we identified relevant studies by searching PubMed and MEDLINE using the following search terms: [(*Staphylococcus aureus* bacteremia OR SAB) OR (methicillin-resistant *Staphylococcus aureus* OR MRSA)] AND [bacteremia] AND [treatment OR predisposing factors OR risk factors]. And relevant English-language, peer-reviewed studies from 2000 to 2026 were included. Two independent investigators (A.K. and C.T.) screened the articles and reviewed the full texts of eligible records. Any disagreements were resolved via discussion and consensus.

## 2. Epidemiology

MRSA is characterized by resistance to beta-lactam antibiotics and cephalosporins (except fifth-generation cephalosporins) mediated by the mecA gene, which produces altered PBP2a protein that makes MRSA resistant to β-lactam antibiotics [6,7]. In addition, the MRSA SCCmec element has the *mecC* gene (a homologous gene of the *mecA* gene), which encodes the PBP2c protein, which, like the PBP2a protein, portends low antibiotic affinity [6,7]. Globally MRSA has been estimated to have a 14.69% colonization rate in elderly populations [8]. MRSA prevalence has been shown to be higher in Africa compared to Asia and South America (22.5% vs. 13.1% vs. 5.4%, respectively), with rates of 0–2% in United States and Europe [9]. MRSA bacteremia is defined as positive blood culture for MRSA [5], often accompanied with fever and systemic signs like hypotension. It is estimated that over 120,000 of these infections occur every year in the United States, leading to 20,000 deaths annually [5].

MRSA bacteremia can occur in conjunction with skin or soft tissue foci/osteoarticular infections/pneumonia or in association with foreign bodies/indwelling catheters/devices. Epidemiologically, MRSA bacteremia can be classified as hospital-acquired (nosocomial); healthcare-associated, community onset; and community-acquired per the seminal study by Friedman et al. [10]. Hospital-acquired bacteremia refers to nosocomial infection, i.e., not present at the time of hospital admission, and developing 48 h or more after admission, usually occurring in association with infected central venous catheters; peripheral intravenous catheters; underlying diabetes mellitus; chronic renal disease; or immunocompromising conditions, including malignancies [10,11,12,13]. Healthcare-associated community-onset MRSA bacteremia refers to MRSA bloodstream infection in patients developing withing 48 h of admission with recent, extensive contact with healthcare, including those with a history of hospitalization within 90 days, dialysis, nursing-home residence, or receipt of intravenous therapy at home [10]. Community-acquired MRSA bacteremia is commonly observed in people without any recent healthcare contact, such as in people who inject drugs, and those with focal MRSA infections, including vertebral osteomyelitis or epidural abscess [4,10].

MRSA bacteremia is associated with high rates of metastatic complications like infective endocarditis (10% to 25%) and other disseminated infections, including vertebral osteomyelitis (4–9%), septic arthritis (7–11%), pneumonia (9%), prosthetic joint infections (30–40%), and meningitis (<1%) [2,6,14,15,16,17,18,19]. MRSA bacteremia is associated with mortality rates of 20–30% [14,19].

Increased mortality rates have been associated with pre-existing comorbidities and early positivity of blood cultures ≤ 12 h [20,21]. MRSA bacteremia is frequently associated with persistent bacteremia of more than 10 days, increased recurrences within 60 days, and higher hospital readmission rates [22].

### 2.1. Predisposing Factors for MRSA Bacteremia

The risk factors for *S. aureus* bacteremia (SAB) [23] frequently overlap with those for MRSA bacteremia; arising from a complex interplay of host, environmental, and behavioral factors. Understanding these risk factors is essential for guiding clinical management, preventing complications, and improving outcomes. These predisposing factors are detailed below.

#### 2.1.1. Indwelling Prosthetic Materials

Implanted foreign materials including intravascular catheters, surgically implanted devices, and orthopedic prostheses are a well-established risk factor for MRSA bacteremia, as they can serve as a nidus for infection once colonized [23].

Intravascular catheters are the most common source of SAB in hospitalized patients and are increasingly recognized as an important cause of community-acquired infection [10,23,24]. These devices provide direct access to the bloodstream, facilitating entry of *S. aureus*. Among patients with cardiac implantable electronic devices, MRSA bacteremia may arise from either a primary device infection or an alternative source. Notably, device involvement is common in this setting, with infection rates reported in approximately 30–50% of cases [23,25]. Newer technologies, such as leadless pacemakers, have emerged as promising alternatives and appear to carry a substantially lower risk of bacterial colonization and infection compared to traditional systems [23,26].

The ability of MRSA to form biofilms plays an important role in medical device-related infections [27]. Biofilm formation involves many stages, including attachment, maturation, and dispersal [28]. Bacterial Microbial Surface Components Recognizing Adhesive Matrix Molecules (MSCRAMMs), mainly clumping factor A (ClfA) and clumping factor B (ClfB) [29], are crucial for attachment to biotic (endovascular) or abiotic (catheter) surfaces [30]. Attachment is followed by maturation of biofilms when they begin to secrete the extracellular polymeric substance (EPS), with polysaccharide intercellular adhesin (PIA), which plays an important role in prevention of phagocytosis, thereby facilitating persistent infection [31].

#### 2.1.2. Nasal Colonization

Patients colonized with *S. aureus* in the nares are more likely to develop bacteremia, and studies have shown 20-fold increased risk of developing MRSA bacteremia if colonized [23,32]. Patients with conditions that increase colonization—such as diabetes or dialysis dependence—are at particularly high risk.

#### 2.1.3. Host-Related Factors

MRSA bacteremia incidence varies by age and gender, with highest rates observed at the extremes of age and among males [33]. Certain populations, including Indigenous communities in Australia and New Zealand, experience disproportionately higher rates, likely reflecting socioeconomic disparities [33]. Chronic medical conditions, particularly diabetes mellitus, dialysis dependence, malignancy, and corticosteroid use, increase MRSA bacteremia risk [23,34,35]. Hemodialysis carries an especially high risk, with incidence up to 100 times higher than in the general population; use of central venous catheters for dialysis access is a key driver [35]. Social determinants of health—including housing instability, limited transportation, and healthcare access—further influence infection risk and outcomes.

#### 2.1.4. Genetic Factors

Evidence supports a genetic contribution to SAB risk. Familial clustering, population-level differences in infection rates, and rare genetic syndromes highlight host susceptibility. Variants in the HLA class II region, particularly near *HLA-DRA* and *HLA-DRB1*, influence immune responses to *S. aureus* and its superantigens, shaping susceptibility to infection and risk of complications, including endocarditis [23,36,37].

#### 2.1.5. Injection Drug Abuse

People who inject drugs (PWIDs) are at high risk for MRSA bacteremia, often presenting at a younger age, without other significant comorbidities [23,38]. High rates of nasal colonization with *S. aureus*, combined with repeated injection of nonsterile materials, substantially increase susceptibility. The colonizing strain often matches the bloodstream isolate, underscoring the direct link between colonization and infection. Substance-use disorders also pose challenges to treatment adherence, emphasizing the need for integrated addiction care, harm-reduction strategies, and close follow-up.

#### 2.1.6. Human Immunodeficiency Virus (HIV) Infection

HIV infection increases MRSA bacteremia risk, independent of injection drug use [23,39]. Low CD4 counts are the strongest predictor of susceptibility, emphasizing the need for careful monitoring and prophylactic strategies in immunocompromised patients.

#### 2.1.7. Risk Factors for Recurrent Infection

Recurrent MRSA bacteremia occurs in a minority of patients. In large cohort studies, about 7% experienced reinfection, typically more than 90 days after the initial episode [23,40]. Risk factors for recurrence include injection drug use, diabetes with complications, paraplegia, severe liver and renal disease, highlighting the interplay of medical, behavioral, and social risk factors in long-term outcomes. 

### 2.2. Pathogenesis of Persistent MRSA Bacteremia

Traditionally, persistent methicillin-resistant *Staphylococcus aureus* (MRSA) bacteremia has been defined as blood cultures remaining positive for 7 days; however, the recent literature has suggested a shorter definition of 2 days of positive blood cultures to encourage earlier intervention [41,42]. Pathogenesis of persistent MRSA bacteremia is not fully understood. Studies have indicated that the (*agr*) system, a quorum-sensing system regulating exotoxins and exoenzyme expression, plays a key role in persistent MRSA bacteremia [43]. MRSA isolates with dysfunctional *agr* system have been associated with higher rates of persistent MRSA bacteremia, as well as higher morbidity and mortality [44,45]. Dysregulated *agr* system is thought to result in upregulated adhesins, leading to greater intracellular invasion and antibiotic tolerance, thus facilitating persistent bacteremia [45,46,47]. The ability of MRSA to form biofilms is also thought to play an important role in host immune evasion and adaptation to survive in inhospitable environments, thereby causing persistent infections [39]. Biofilms, because of their structure and polymeric matrix, provide a protective environment by preventing diffusion of antibiotics and, in addition, protect against environmental stressors, thus playing a critical role in persistent infections [44,45,46].

## 3. Management of MRSA Bacteremia

### 3.1. Diagnostic Considerations

Even a single blood culture positive for MRSA is considered significant [48]. If a blood culture is positive for MRSA, empiric antibiotic treatment needs to be initiated immediately with clinical evaluation, including a thorough history and physical examination. Blood cultures need to be repeated in 24–48 h to demonstrate clearance of bacteremia [41,49]. Failure to clear blood cultures despite treatment is associated with poor prognosis, and patients with persistent bacteremia have been shown to have higher 30-day mortality rates than those with immediate clearance of bacteremia [49]. All patients with MRSA bacteremia should be evaluated for infective endocarditis (IE), and echocardiography should be performed [41]. Infectious disease consultation for all cases of MRSA bacteremia is recommended [50]. The need for additional studies, e.g., abdominal CT, head imaging, bone MRI, etc., is determined by clinical presentation, complications, and metastatic involvement [41].

### 3.2. MRSA Bacteremia: Approach to Treatment

The Infectious Disease Society of America (IDSA) guidelines recommend determining anti-MRSA antimicrobial therapy based on whether the bacteremia is considered uncomplicated or complicated [41]. Uncomplicated bacteremia is defined as positive MRSA blood culture results, without endocarditis, no implanted prosthesis, lack of continued growth on blood cultures obtained 2–4 days after initial therapy, and if the patient is afebrile within 72 h of therapy initiation. Complicated bacteremia includes patients with positive blood culture and any of the following: endocarditis, an implanted prosthesis, continued positive blood cultures, or sustained pyrexia after 72 h of therapy [41].

According to the Infectious Disease Society of America (IDSA) guidelines, treatment of MRSA bacteremia consists of source control and use of appropriate antimicrobials [41]. Source control entails removal of foreign bodies and indwelling devices, and draining purulent debris/fluid collections. As noted previously, intravascular catheters and indwelling devices, including surgical implants/prostheses, are important predisposing factors for MRSA bacteremia, and removal of infected foreign material is crucial for clinical resolution. Complicated MRSA bacteremia usually requires better source control, longer duration of therapy, and closer follow-up.

Antibiotic treatment of MRSA bacteremia includes monotherapy and combination antibiotic therapy regimens. Vancomycin and daptomycin are preferred antibiotic monotherapy agents for treatment of MRSA bacteremia [41,51]. Alternative monotherapy agents include ceftaroline, ceftobiprole, lipoglycopeptides (dalbavancin, oritavancin, and telavancin), linezolid, and tedizolid. The various anti-MRSA antimicrobials are presented in Table 1 [41,51,52,53,54,55]. Both adult and pediatric dosing of the antimicrobials are described in Table 2 [41,51,52,53,54,55].

### 3.3. Antimicrobial Therapy: Guidelines for Treatment of MRSA Bacteremia

IDSA recommendations for the choice of antimicrobials for both uncomplicated and complicated MRSA bacteremia are similar, recommending the utilization of vancomycin or daptomycin as a first-line therapy option [41].

Vancomycin has a long-standing history of being a first-line agent for MRSA bacteremia despite the risk of toxicity and dosing drawbacks. Historically, vancomycin dosing strategies relied on serum trough concentrations, typically 10–20 mg/L, with target selection adjusted according to infection severity and clinical context [41]. However, accumulating evidence demonstrates that trough-based monitoring is associated with a higher incidence of vancomycin-induced acute kidney injury compared with dosing strategies guided by the area under the concentration (AUC) [56,57]. In response, recent clinical practice guidelines now advocate for AUC-guided dosing as the preferred method for optimizing therapeutic efficacy while minimizing nephrotoxicity [54]. Therapeutic monitoring of vancomycin was recently revised according to the 2020 consensus guidelines, and a reduction of vancomycin exposure and kidney injury was reported when the use of AUC/MIC (minimum inhibitory concentration) monitoring was employed in place of serum trough monitoring [54]. AUC-guided vancomycin dosing is likewise recommended for pediatric patients, as reflected in the 2020 IDSA consensus guidelines, which were co-authored by the Pediatric Infectious Diseases Society and endorse an AUC/MIC target of 400–600 mg·h/L for serious MRSA infections [54].

Dose adjustment of vancomycin is guided by area under the curve over 24 h to minimum inhibitory concentration (AUC/MIC) monitoring with a goal of 400 to 600 mg·hr/L. Factors such as patient weight, renal function, body surface area (BSA), and drug interactions are taken into consideration when the initial dose is calculated, and when subsequent dose adjustments are needed [54,55,56,57]. Periodic monitoring of AUC and renal function helps reduce the risk of toxicity and acute kidney injury.

In cases of uncomplicated bacteremia, the literature recommends dosing vancomycin to achieve a goal area under the curve (AUC)/MIC value of around 400 to maximize therapeutic effect and minimize the potential for nephrotoxicity. In cases of complicated bacteremia, a vancomycin AUC/MIC value of 400–600 is considered appropriate, recognizing that as an AUC/MIC of 600 is approached, the increased potential for nephrotoxicity exists [54]. These AUC/MIC values assume an MIC of ≤1 mg/L. In MRSA isolates with an MIC > 1 mg/L, therapeutic efficacy with vancomycin is difficult to achieve, and alternative therapies may be considered. Although the IDSA guidelines advocate minimum inhibitory concentration (AUC/MIC) monitoring, there are important practical considerations regarding implementation of vancomycin AUC/MIC monitoring. Adoption varies across institutions [58] influenced by local laboratory and pharmacy resources, and a study showed that the majority of US institutions had not fully integrated these methods into their practice [58]. Although broth microdilution (BMD) is the preferred method for measuring MIC, laboratories often lack the capacity for it. To address this, the guidelines allow for a pragmatic practice: assuming an MIC of 1 mg/L based on typical local susceptibilities, thereby simplifying the target to an AUC range of 400–600 [59,60]. Widely adopted current practice routinely uses broth microdilution (BMD) to determine vancomycin MIC values, consistent with IDSA recommendations identifying BMD as the reference standard and the AUC calculator assumes an MIC of 1 mg/L, which aligns with guideline-supported pragmatic practice. This assumption is necessary because vancomycin is typically initiated empirically, prior to availability of organism-specific MIC data, requiring an MIC estimate for initial pharmacokinetic calculations. Some programs use Monte Carlo models and population kinetics to predict AUC, which is a flexible method requiring only one trough level at steady state to produce an accurate estimate. However, cost and resources are the most significant deterrents for many institutions. If this software is unavailable in local settings, the pharmacists use Pharmacokinetic (PK) Equations that would need both the vancomycin peak and trough levels to estimate the AUC [59,60,61], thus creating a substantial burden for phlebotomy and nursing, as well as patient discomfort. To circumvent this, many local institutions, including ours, do not rely on a Monte Carlo prediction model or routine collection of peak and trough concentrations but utilize an internally developed AUC calculator [59] based on pharmacokinetic data derived from an institutional patient population. This model estimates vancomycin clearance and calculates the total daily dose required to achieve a specified AUC. Subsequent AUCs and dose adjustments are estimated using midpoint concentrations within this framework, thereby minimizing the need for multiple blood draws and reducing patient inconvenience. This approach allows for practical and sustainable implementation of IDSA-recommended AUC-guided vancomycin dosing while maintaining dosing accuracy and minimizing workflow burden.

Daptomycin is another first-line treatment option which is also bactericidal like vancomycin and can be considered an alternative to vancomycin for MRSA bacteremia when the vancomycin MIC is 1.5–2.0 mg/L [41,51,53]. Daptomycin dosing is less aggressive in uncomplicated MRSA bacteremia, with a recommended dose of 6 mg/kg IV daily. In patients with complicated MRSA bacteremia, doses of 8–10 mg/kg IV daily may be considered [41,53]. Caution should be taken when switching from vancomycin to daptomycin when the vancomycin MIC > 1.0 mg/L. As there are higher rates of non-susceptibility daptomycin and clinical failures, checking daptomycin MICs in this scenario is recommended. Daptomycin MIC ≤ 1 mg/L is generally considered susceptible, and poorer outcomes have been associated with higher MICs. Like vancomycin, daptomycin has weight-based dosing and needs to be adjusted based on renal function. However, unlike vancomycin, drug levels are unnecessary to make subsequent dose adjustments. Monitoring of creatine phosphokinase (CPK) levels should be done at least weekly or more frequently if the patient is on concurrent statin therapy, as there is a risk of myopathy and rhabdomyolysis [41,52,53]. Discontinuation is recommended in patients with symptomatic myopathy and CPK ≥ 5 times the upper limit of normal (ULN) or in asymptomatic patients with CPK ≥ 10 times ULN.

Daptomycin’s lack of efficacy in treating respiratory infections prevents it from being a universal substitute or equivalent for vancomycin. Daptomycin is rapidly inactivated by pulmonary surfactant, and surfactant molecules physically trap the antibiotic, preventing it from reaching and disrupting the bacterial cell membranes in the alveoli [62]. Due to this inactivation by pulmonary surfactant, daptomycin failed to meet non-inferiority criteria in clinical trials for community-acquired pneumonia and is not recommended for treatment of pulmonary infections [62].

IDSA guidelines were last updated in 2011, and more recent European guidelines published in 2021 in the United Kingdom (UK) have modified the uncomplicated MRSA therapy recommendations from vancomycin and daptomycin alone to offering additional options with linezolid or teicoplanin (not approved for use in the United States) [51]. European and UK guidelines take a more cautious, stewardship-focused approach to managing MRSA and place strong emphasis on treating based on the type of infection. Although several newer anti-MRSA antibiotics are now available, these guidelines acknowledge that the evidence is still limited, and many recommendations are based on clinical experience rather than strong head-to-head trials.

One key difference from US guidance is the focus on matching treatment with severity and location of infection, rather than following a strict ranking of antibiotics. Across all cases, source control is essential, and glycopeptides, mostly vancomycin (or teicoplanin in Europe), remain first-line for most serious infections [51]. There is also a strong emphasis on avoiding unnecessary antibiotics, especially in milder cases, and involving multidisciplinary teams for more complex infections.

For MRSA bacteremia, vancomycin is typically used first, with daptomycin, linezolid, or teicoplanin used as alternatives when needed. Treatment usually lasts about 2 weeks for uncomplicated cases and 4 weeks or longer for complicated infections. In endocarditis, vancomycin remains standard, with daptomycin used as an alternative, sometimes in combination for more difficult cases. These guidelines are generally more cautious about using combination therapy early, reflecting uncertainty in the evidence [51].

First-line therapy remains vancomycin, with similar goal AUC values as mentioned in the IDSA Guidelines, but if contraindicated, linezolid is considered an alternative first-line agent. Linezolid and tedizolid are oxazolidinones that can be used as alternative therapy and may have enhanced efficacy against strains of MRSA that produce toxins, such as Panton–Valentine leucocidin, alpha-hemolysin, and toxic shock syndrome toxin 1. A few of the benefits of linezolid include that it can be given either intravenously or orally, has excellent tissue distribution, and does not need to be renally adjusted [41,53,54,55].

Additionally, linezolid provides an effective oral therapeutic alternative that can facilitate earlier hospital discharge and eliminate the need for outpatient parenteral antimicrobial therapy, including agents such as vancomycin or daptomycin. Monitoring parameters should include labs values such as CPK and weekly CBCs. Signs and symptoms of rhabdomyolysis, thrombocytopenia, and serotonin syndrome should be monitored as well. Linezolid is a known reversible monoamine oxidase inhibitor and thus interacts with serotonergic agents, increasing the risk of serotonin syndrome. Due to this increased risk, many serotonergic agents have not been recommended for concurrent use with linezolid. However, emerging evidence indicates that the risk of serotonin syndrome associated with linezolid is substantially lower than previously believed. Recent systematic reviews demonstrate an extremely low incidence of serotonin toxicity with linezolid, both as monotherapy and when co-administered with serotonergic agents, suggesting that prior concerns may have overstated the true clinical risk [63].

The European UK guidelines continue to support using linezolid as an alternative first-line agent, daptomycin as a second-line agent, and additionally teicoplanin, where available [51].

Teicoplanin, where available, has been considered preferred monotherapy [51]. It has a similar spectrum of activity as vancomycin; however, tolerability is improved. To maintain therapeutic levels, weekly monitoring of serum trough concentrations with potential dose adjustments is recommended. Renal function monitoring and auditory testing are warranted, as teicoplanin carries the risk of acute renal impairment or failure, and ototoxicity, which increases with concomitant use of other nephrotoxic and ototoxic medications. Overall, compared to IDSA-based approaches, UK and European guidelines are more measured and conservative, focusing on individualized care, careful antibiotic use, and recognizing the limits of the current evidence [51].

### 3.4. Duration of Antimicrobial Therapy

Length of therapy must also be considered when treating both classifications of bacteremia. Both the IDSA Guidelines and the UK consensus recommend treating uncomplicated bacteremia for a minimum of 2 weeks [41,51]. Complicated bacteremia requires a treatment duration of 4–6 weeks depending on the extent and resolution of the infection. Once treatment is initiated, guidelines recommend repeating blood cultures for documenting clearance of bacteremia.

## 4. Recent Developments

MRSA bacteremia requires a multipronged approach involving the removal of infected foci coupled with timely initiation of appropriate antimicrobial therapy. Persistently positive blood cultures after initiation of treatment for MRSA bacteremia are associated with high fatality and continue to pose a clinical challenge [42,49]. Failure to clear blood cultures despite treatment is associated with poor prognosis, and patients with persistent bacteremia have been shown to have a greater risk of dying than those with immediate clearance of bacteremia [42], with studies noting mortality rates of 10% at 7 days and about 25% at 3 months [19,42]. Numerous studies have recently investigated treatment options for MRSA bacteremia to address this clinical challenge [42,64,65,66,67,68,69,70,71,72,73,74,75,76]. Evaluation for metastatic complications (i.e., endocarditis and paravertebral abscess) should be the next step before considering switching antibiotics/adding another antibiotic [41]. Notwithstanding advancements in the field, managing persistent MRSA bacteremia remains a subject of expert debate. Vancomycin or daptomycin remain the antibiotics of choice for monotherapy per the guidelines [41,51], and the recent literature suggests tailoring antibiotic strategies with the addition of a second agent, such as ceftaroline, for persistent bacteremia [42,70,71,72,73,74,75,76].

Recent developments, including newer cephalosporins, long-acting dalbavancin, combination antibiotic regimens and evaluation of adjunctive treatments like bacteriophage lysins, as well as gaps in knowledge and current practices for managing persistent MRSA bacteremia, are discussed below.

### 4.1. Newer Cephalosporins

Ceftaroline and ceftobiprole are fifth-generation broad-spectrum cephalosporins that are active against MRSA (Table 1 and Table 2) [51,52,53,55,66,67]. Both medications are relatively newer, with ceftaroline being FDA approved in 2010 and ceftobiprole in 2024. Both are administered in prodrug formulations, ceftaroline fosamil and ceftobiprole medocaril, and then rapidly metabolize to their active formulations, ceftaroline and ceftobiprole, respectively. Ceftaroline use in MRSA bacteremia has been widely reported but remains an off-label indication; and ceftobiprole has shown promising results in treating MRSA bacteremia [42,64,67,68,69,70,71]. In a double-blind phase 3 clinical trial (ERADICATE Clinical Trial), ceftobiprole was shown to be non-inferior to daptomycin for treating complicated SAB, including MRSA bacteremia [68].

Numerous studies have reported that the addition of ceftaroline to vancomycin or daptomycin is an effective salvage therapy for complicated, persistent MRSA bacteremia [70,71,72,73,74,75,76]. Although the exact mechanism of synergy remains unclear, it likely involves the simultaneous binding and inhibition of multiple Penicillin-Binding Proteins (PBPs), such as PBP 1, 2, and 4, which can lead to more rapid cell-wall disruption [77]. Furthermore, beta-lactams may enhance the penetration of drugs like vancomycin or daptomycin by thinning the cell wall and altering the bacterial surface charge [77]. The inverse relationship between glycopeptide and β-lactam susceptibilities is known as the “see-saw effect” [77]. In the CAMERA-2 trial, adding a beta-lactam to vancomycin was shown to significantly reduce the duration of bacteremia and lead to faster clearance (median 56 h vs. 66 h); however, the combination was shown to have greater risk of acute kidney injury [78].

Both ceftaroline and ceftobiprole require periodic monitoring of renal function, with dose adjustments as necessary [52,53,55]. Monitoring for signs and symptoms of neurotoxicity in patients taking either ceftaroline or ceftobiprole is recommended, as encephalopathy and seizures have occurred with increasing risk in patients with kidney impairment. In addition, patients receiving prolonged ceftaroline use for >7 days should have hematologic parameters followed to assess for neutropenia and anemia. Prolonged use of either agent may result in fungal or bacterial superinfections, including *Clostridioides difficile* diarrhea. Both fifth-generation cephalosporins have shorter half-lives, ceftaroline approximately 1–3 h and ceftobiprole 3 h, which allows for dosing every 6 to 8 h depending on the medication [52,53,55].

### 4.2. Long-Acting Lipoglycopeptide Antimicrobials

Dalbavancin, oritavancin, and telavancin are all intravenous lipoglycopeptide antibiotics that are unique in that they do not need to be dosed multiple times per day [52,53,55]. Dalbavancin and oritavancin are considered long acting, as they have half-lives of 14 and 10 days, respectively, and only need to be dosed once or weekly (Table 1 and Table 2). This can be advantageous when treating patients that refuse inpatient treatment and/or have long-term intravenous access that is not preferred due to risk of complications or drug-seeking behaviors.

While vancomycin or daptomycin monotherapy remain the drugs of choice for empiric treatment of MRSA bacteremia, a recent clinical trial (“Dalbavancin for Treatment of *Staphylococcus aureus* Bacteremia: The DOTS Randomized Clinical Trial”) has shown encouraging results with two weekly 1500 mg doses of dalbavancin (on days 1 and 8), with potential advantages over extended daily intravenous antibiotic therapy that could obviate the need for PICC lines and need for daily infusions for MRSA bacteremia [65].

Telavancin has a half-life of 7 h and would need to be continued past 1 day of therapy, unlike the other two agents [52,53,55]. Renal function and infusion-related reactions should be monitored in all three agents. Additionally, liver function tests should be monitored with dalbavancin, and signs and symptoms of osteomyelitis with oritavancin. Pregnancy status should be verified in females of reproductive potential prior to use of telavancin, as it may cause fetal harm. Effective contraceptive use is recommended during treatment and for at least 2 days post-treatment end date.

### 4.3. Combination Therapy

The use of combination therapy has emerged within the past few years as salvage therapy for refractory MRSA bacteremia. Daptomycin plus ceftaroline, daptomycin plus a beta-lactam (including cephalosporins, piperacillin/tazobactam, and ampicillin/sulbactam), vancomycin plus a hydrophilic beta-lactam (i.e., cefazolin and ceftaroline) are a few examples of these combination regimens.

Several studies have reported successful use of combination therapy in MRSA bacteremia [42,70,71,72,73,74,75,76]. For persistent bacteremia, it has been recommended that if repeat blood cultures fail to become negative at 3–5 days despite appropriate antimicrobial therapy, it is considered to be a failure of monotherapy, and addition of ceftaroline to vancomycin or changing to daptomycin with a second antibiotic agent should be considered [56,64]. High-dose daptomycin (with a second agent to prevent daptomycin-resistance) and addition of ceftaroline have been considered the best practice currently for persistent MRSA bacteremia [42]. A survey-based study noted that over half of the clinicians reported that they would switch to daptomycin or daptomycin with ceftaroline in case of persistent MRSA bacteremia [79]. There are potential risks when implementing combination therapy, as vancomycin plus a hydrophobic beta-lactam (i.e., oxacillin, flucloxacillin, and cloxacillin) increases the risk of nephrotoxicity [80]. Increased risk of renal toxicity has been noted when combining vancomycin with rifampin or vancomycin with gentamicin [41]. The value of using vancomycin plus rifampin for MRSA prosthetic infections is often debated. In the presence of prosthetic material, rifampin is frequently added for its activity against biofilm-associated organisms; however, it should never be used as monotherapy, due to rapid resistance emergence [81]. While rifampin is often recommended for prosthetic/device-associated infections due to its activity against biofilm, and may be beneficial in selected cases with retained hardware, its use should be individualized given concerns about drug interactions, hepatotoxicity, and rapid resistance when used inappropriately. Rifampin is not universally required and should be reserved for carefully selected patients.

Another controversy is related to the questionable need of aminoglycosides for MRSA endocarditis and increased risk of nephrotoxicity thereby, potentially causing more harm than benefit. Accumulating evidence indicates that the addition of aminoglycosides (e.g., gentamicin) provides minimal-to-no mortality benefit in MRSA endocarditis, while significantly increasing the risk of nephrotoxicity [82,83]. More recent studies and guideline updates have moved away from routine use of aminoglycosides in this setting, and aminoglycosides are not recommended in native valve endocarditis, apart from brief use in prosthetic valve endocarditis [41].

Also, the addition of gentamicin or rifampin has not been shown to enhance the effectiveness of daptomycin in the treatment of experimental endocarditis due to MRSA [84]. It was shown that adding these antibiotics may only increase the risk of side effects, without providing additional benefit in clearing bacteria or success in treating the infection [84].

Other combination regimens with limited data include daptomycin plus fosfomycin, daptomycin plus trimethoprim/sulfamethoxazole, and ceftaroline plus trimethoprim/sulfamethoxazole. Prior to implementing any of the above combination regimens, patient risk factors and potential adverse effects need to be taken into consideration.

Prolonged or continuous infusion of vancomycin is another strategy that has been proposed to combat serious infections. Prolonged or continuous infusion of vancomycin (CIV) often reaches the desired therapeutic steady-state concentration faster than intermittent dosing, thereby increasing the time above minimum inhibitory concentration [85,86]. Studies indicate that CIV is associated with a lower risk of acute kidney injury (AKI) compared to intermittent infusion. Thus, prolonged infusion might be a strategy to consider for persistent MRSA bacteremia, although large clinical trial data and consensus guidelines are lacking at this point.

### 4.4. Molecular and Clinical Determinants of Targeted Therapy in MRSA

While treatment of MRSA bacteremia is primarily guided by clinical syndrome and antimicrobial susceptibility testing, emerging evidence suggests that certain molecular subtypes and resistance phenotypes may influence antimicrobial selection and therapeutic response. However, it is important to note that routine clinical decision-making is still largely phenotype- and syndrome-driven, as rapid molecular stratification is not yet universally actionable at the bedside. MRSA strains are broadly categorized into community-associated (CA-MRSA) and healthcare-associated (HA-MRSA) lineages, which differ in virulence factors and resistance patterns [87,88,89].

CA-MRSA strains, particularly the USA300 (ST8) lineage, frequently harbor Panton–Valentine leukocidin (PVL) and are associated with severe skin and soft tissue infections and necrotizing pneumonia. These isolates often retain susceptibility to non-β-lactam agents such as trimethoprim–sulfamethoxazole, doxycycline, and clindamycin. In toxin-mediated disease (e.g., necrotizing pneumonia), agents that suppress toxin production, such as linezolid or clindamycin, may offer theoretical and clinical advantages [87].

In contrast, HA-MRSA strains (e.g., ST5 and ST239) typically demonstrate broader multidrug resistance and are more commonly associated with invasive infections such as bacteremia and device-related infections. Glycopeptides (vancomycin) and lipopeptides (daptomycin) remain first-line therapies in these cases [88,89].

Specific resistance phenotypes further influence treatment selection. Vancomycin-intermediate S. aureus (VISA) is associated with cell-wall thickening and reduced glycopeptide susceptibility, often resulting in clinical failure. In such cases, high-dose daptomycin—frequently in combination with a β-lactam—has emerged as a preferred strategy.

Clinical syndrome remains the most important determinant of therapy for MRSA [51]. For instance, for skin and soft tissue infections, simple drainage is often enough for smaller abscesses, and antibiotics are reserved for more severe or high-risk situations. When antibiotics are needed, oral options, including clindamycin or co-trimoxazole, are used, while more serious infections require intravenous therapy [51]. Bone and joint infections typically need both surgery and long courses of antibiotics. Treatment often starts with intravenous therapy and may transition to oral agents when appropriate. Rifampin is used in combination (and never alone), especially when prosthetic material is involved [51]. For MRSA pneumonia, vancomycin or linezolid is recommended, with linezolid often preferred in more severe cases because it reaches lung tissue well and may help reduce toxin effects [51]. Although certain molecular features (e.g., PVL production and VISA phenotype) can inform antimicrobial selection in specific contexts, most MRSA treatment decisions remain driven by infection type, severity, and clinical response rather than genotype alone. The integration of rapid molecular diagnostics into therapeutic decision-making remains an important area for future investigation.

### 4.5. Novel Potential Treatment Strategies

Other treatment strategies may have a potential role as adjunctive agents in treatment of MRSA bacteremia. Novel treatment strategies, including bacteriophage-derived lysins that eradicate MRSA biofilms, offer promise as adjunctive therapies with antibiotics. Exebacase (Lysin CF-301), a bacteriophage-derived lysin, was shown to have bacteriolytic activity against MRSA and was noted to be synergistic with vancomycin and daptomycin in vitro, with improved survival in staphylococcal bacteremia in a murine model [90,91,92]. However, in a phase 3 trial (“Exebacase in Addition to Standard-of-Care Antibiotics for *Staphylococcus aureus* Bloodstream Infections and Right-Sided Infective Endocarditis: A Phase 3, Superiority-Design, Placebo-Controlled, Randomized Clinical Trial (DISRUPT)”), exebacase in combination with antibiotics failed to show a favorable clinical response in patients with MRSA bacteremia/endocarditis at day 14 [93].

Another endolysin-based candidate SAL200 using SAL-1, a bacteriophage derived endolysin, has been shown to be synergistic with antibiotics in vitro and in a murine model [94,95,96]. Other agents under investigation include 514G3, a monoclonal antibody that acts by binding to staphylococcal protein A (SpA), thereby evading the immune system [97]. In mice, 514G3 was found to have synergy with vancomycin and was associated with increased survival six days after bacterial challenge in a bacteremia model [98].

Additionally, vaccine candidates for *Staphylococcus aureus* have been investigated. These include rFSAV (composed of five recombinant *S. aureus* antigens (Hla, SEB, MntC, IsdB, and SpA)) undergoing clinical trials [99], and IBT-V02 (consisting of seven *S. aureus* toxoids), which has been shown to be effective in pre-clinical studies [100]. Other therapeutic agents targeting MRSA biofilms, including nanoparticles (gold, silver, iron, copper, and selenium), laser shock waves, phytochemicals, and antimicrobial peptides, have been proposed as adjunctive treatment strategies for MRSA infections [101,102,103,104,105]. However, the role of these agents is largely experimental, and further clinical studies are needed for potential application in MRSA bacteremia.

## 5. Psychological and Behavioral Considerations

Additionally, socio-psychological aspects also need consideration, as psychological and behavioral factors significantly influence outcomes in MRSA bacteremia/related complications. Substance-use disorders, especially injection drug use, are strongly associated with both incident and recurrent MRSA bacteremia [106,107,108]. These patients experience higher rates of nonadherence, patient-directed discharge, and reinfection. Integration of addiction treatment, including medications for opioid-use disorder, into infection management improves engagement and clinical outcomes [109]. Depression and anxiety are common in medically ill populations and are associated with reduced adherence, impaired self-care, and increased healthcare utilization [110]. In the setting of serious infection, these factors may contribute to missed antimicrobial doses, poor follow-up, and worse clinical outcomes. Severe mental illness, including symptoms such as avolition, may further impair the ability to adhere to treatment, particularly when regimens are prolonged or complex [111]. Cognitive impairment and limited health literacy also contribute to unintentional nonadherence and should be addressed through simplified communication and care planning [112]. Social determinants of health, including unstable housing, limited transportation, and reduced access to care, are important contributors to treatment interruption and adverse outcomes [113]. In addition, stigma and mistrust of the healthcare system may reduce engagement, particularly among patients with substance-use disorders [114]. Recognition of these factors and incorporation of multidisciplinary care, including addiction medicine, behavioral health, and social support—are essential to optimize adherence and improve outcomes in MRSA bacteremia.

## 6. Conclusions

MRSA bacteremia, given its high morbidity and mortality, requires a comprehensive and coordinated management approach. Effective treatment hinges on prompt source control, through removal or drainage of infected foci, combined with early initiation of appropriate antimicrobial therapy. Vancomycin and daptomycin remain the cornerstone agents for initial monotherapy. Treatment should be guided by clinical response and clearance of bacteremia, with search for metastatic foci and consideration of combination therapy such as the addition of ceftaroline in cases of persistent infection. Even with advances in treatment, persistent MRSA bacteremia continues to pose significant clinical challenges. Emerging therapies, including long-acting agents such as dalbavancin and newer cephalosporins like ceftobiprole, have shown encouraging results in selected settings. In addition, novel approaches such as bacteriophage therapy are under active investigation as potential adjunctive strategies. At present, source control and targeted antibiotic therapy remain the foundation of care. Ongoing research is needed to better define the role of newer agents, optimize combination regimens, and evaluate adjunctive therapies aimed at improving outcomes in this complex and high-risk infection.

## Figures and Tables

**Table 1:** Antimicrobials for treatment of MRSA infections [41,51,52,53,54,55].

Medication	Class	Mechanism of Action	Route	Adverse Drug Reaction	Pharmacokinetics
Ceftaroline	Cephalosporin, 5th generation	Binds to penicillin-binding proteins, which inhibit bacterial cell-wall synthesis.	IV	Common: Rash, fever, diarrhea, nausea/vomiting. Serious: *Clostridioides difficile* diarrhea, elevation in ALT (alanine aminotransferase), anaphylaxis, encephalopathy, seizure.	Absorption: IV 100%. Distribution: Widely distributed. Vd: 20.5 L. Metabolism: Phosphatase enzyme metabolizes to active drug in plasma. Excretion: Renal 88%, fecal 6%, dialyzable. Elimination half-life: 1–3 h.
Ceftobiprole	Cephalosporin, 5th generation	Binds to penicillin-binding proteins, which inhibit bacterial cell-wall synthesis.	IV	Common: Diarrhea, nausea/vomiting. Serious: Hypersensitivity reaction.	Absorption: IV 100%. Distribution: Plasma protein binding 16%. Vd: 18 L. Metabolism: Ceftobiprole medocaril sodium (prodrug) is rapidly metabolized to ceftobiprole (active drug), which undergoes minimal metabolism. Excretion: Renal 89% (83% active drug, 5% inactive drug, <1% unchanged prodrug), dialyzable. Elimination half-life: 3.3 h.
Daptomycin	Cyclic lipopeptide	Binds to bacterial cell membranes and causes cell death by inducing rapid depolarization of the membrane potential, leading to inhibition of intracellular DNA, RNA, and protein synthesis.	IV	Common: Hypertension, hypotension, pruritus, rash, abdominal pain, diarrhea, vomiting, dizziness, headache, insomnia, dyspnea, pain in throat, fever. Serious: Increased CK, disorder of muscle, rhabdomyolysis, renal failure, pulmonary eosinophilia, disease caused by Gram-negative bacteria.	Absorption: IV 100%. Distribution: 92% protein binding. Widely distributed. Inactivated by lung surfactants. Minimal CNS penetration. Vd: 0.1 L/kg Metabolism: Minimal Excretion: Renal 78% (unchanged drug), fecal 20% (unchanged drug), dialyzable. Elimination half-life: 8 h adults, 4.4–7.5 h pediatrics.
Delafloxacin	Fluoroquinolone	Inhibits DNA replication, repair, recombination, and transcription by inhibiting DNA topoisomerases.	IV, PO	Common: Diarrhea, nausea/vomiting, ALT/SGPT elevation, AST elevation, headache. Serious: Aortic aneurysm or dissection, hypoglycemia, hypersensitivity reaction, myasthenia gravis with exacerbation, rupture of tendon, tendonitis, disoriented, disturbance of attention, memory impairment, peripheral neuropathy, pseudotumor cerebri, raised intracranial pressure, seizure, agitation, delirium, feeling nervous, psychotic disorder.	Absorption: IV 100%, Oral 58.8% Distribution: Extensive in skin and lung. Protein binding 84%. Vd: 30–48 L Metabolism: Hepatic via glucuronidation. Excretion: IV = Renal 65%, Fecal 28%. PO = Renal 50%, Fecal 48%. Dialyzable 19%. Elimination half-life: IV 3.7 h, PO 4.2- 8.5 h
Dalbavancin	Glycopeptide	Inhibits bacterial wall synthesis by binding to D-alanyl-D-alanine and blocking glycopeptide polymerization.	IV	Common: Diarrhea, nausea, fever, headache. Serious: Hypersensitivity reaction, elevated ALT/SGPT, *Clostridioides difficile* infection, gastrointestinal hemorrhage.	Absorption: IV 100%. Distribution: Extensive in skin Vd: 9 L. Metabolism: Minor. Excretion: Renal 33% unchanged drug and 12% as metabolite, fecal 20%, non-dialyzable. Elimination half-life: 346 h.
Oritavancin	Glycopeptide	Binds to stem peptides of peptidoglycan precursors, inhibiting cell-wall biosynthesis.	IV	Common: Nausea/vomiting, abscess, headache. Serious: *Clostridioides difficile* infection, infusion reaction, cellulitis, osteomyelitis, hypersensitivity reaction.	Absorption: IV 100%. Distribution: Extensive in skin; Vd indicates extensive distribution into tissues. Vd: 87.6 L. Metabolism: None. Excretion: Renal 5%, fecal 1% (unchanged drug), non-dialyzable. Elimination half-life: 245 h.
Teicoplanin (not available in US)	Glycopeptide	Inhibits bacterial wall synthesis by binding to D-alanyl-D-alanine and blocking glycopeptide polymerization.	IM, IV, PO	Common: Erythema, pruritus, rash, fever. Serious: Stevens–Johnson syndrome, toxic epidermal necrolysis, thrombocytopenia, hypersensitivity reaction, toxic deafness, renal failure.	Absorption: IM > 90%, IV 100%. Distribution: Extensive in skin, blister fluid, myocardium, pulmonary tissue, pleural/bone/synovial fluid. Not readily distributed into CSF. Vd: 0.7–1.4 L/kg. Metabolism: Minor, approximately 3%. Excretion: Renal 80% (unchanged), fecal 2.7%, non-dialyzable. Elimination half-life: Adults 100–170 h, neonates 40 h, pediatrics 58 h
Telavancin	Glycopeptide	Inhibits bacterial wall synthesis by binding to D-alanyl-D-alanine and blocking glycopeptide polymerization. Additionally, disrupts membrane potential and cell permeability.	IV	Common: Abnormal urine, Altered sense of taste, nausea/vomiting. Serious: Acute renal failure, anaphylaxis, prolonged QT interval.	Absorption: IV 100%, Distribution: Extensively through skin and pulmonary tissues. Vd: 0.13 L/kg. Metabolism: Minimal, unknown mechanism. Excretion: Renal 76%, dialysis effects on drug not studied (not recommended). Elimination half-life: 7 h.
Vancomycin	Glycopeptide	Inhibits bacterial wall synthesis by binding to D-alanyl-D-alanine and blocking glycopeptide polymerization.	IV, PO, Rectal	Common: Hypokalemia, abdominal pain, diarrhea, nausea/vomiting Serious: Hypotension, *Clostridioides difficile* diarrhea, agranulocytosis, neutropenia, thrombocytopenia, anaphylaxis, ototoxicity, nephrotoxicity.	Absorption: IV 100%. Oral/rectal: Negligible. Distribution: Widely distributed. Crosses blood–brain barrier. Vd: 0.2 L/kg to 1.25 L/kg. Metabolism: None. Excretion: IV = Renal 40–100% (unchanged drug), PO = fecal extensive (unchanged drug), dialyzable. Elimination half-life: 4 to 6 h in healthy adults; pediatrics 5 to 21 h.
Tigecycline	Glycycline	Inhibits protein synthesis by binding with ribosomal subunits.	IV	Common: Abdominal pain, diarrhea, nausea/vomiting, headache. Serious: Septic shock, *Clostridioides difficile* infection, acute pancreatitis, ALT/SGPT level raised, AST increased, drug-induced disorder of liver, hyperbilirubinemia, liver failure, anaphylaxis, pseudotumor cerebri, all-cause death, sepsis.	Absorption: IV 100%. Distribution: Protein binding 71–89%. Extensive in tissues. Distributes into gallbladder, lung, colon. Unlikely to reach therapeutic levels in CNS. Vd: Adults 7–9 L/kg, age 8–11 years 2.84 L/kg. Metabolism: Hepatic, not extensively metabolized. Excretion: Biliary/fecal 59%, renal 33%, non-dialyzable. Elimination half-life: Single dose 27 h, steady-state 42 h.
Clindamycin	Lincosamide	Inhibits bacterial protein synthesis by binding to rRNA of the 50S subunit.	IV, PO, topical, vaginal	Common: Morbilliform eruption, constipation, backache, headache, urinary tract infectious disease, Candida vaginitis. Serious: Erythema multiforme, Stevens–Johnson syndrome, toxic epidermal necrolysis, *Clostridioides difficile* infection, anaphylaxis, drug reaction with eosinophilia and systemic symptoms, hypersensitivity reaction, gasping for breath.	Absorption: IV 100%, PO 90%, vaginal cream 5%, vaginal suppository 30%. Distribution: Protein binding 94%. Widely distributed in fluids and tissue, including bone. No significant levels in CSF. Vd: 0.6–1.2 L/kg. Metabolism: Extensive in liver and intestine via CYP3A4. Excretion: Renal 10% (unchanged drug), fecal 3.6% (unchanged drug), non-dialyzable. Elimination half-life: IV/IM 2.5–3 h, vaginal cream 1.5–2.6 h, vaginal suppository 11 h.
Fosfomycin	Miscellaneous	Inhibits bacterial wall synthesis by inactivating pyruvyl transferase enzyme which is needed by bacteria for cell-wall synthesis.	IV (not available in US), PO	Common: Diarrhea, nausea, backache, headache, dysmenorrhea, pharyngitis, rhinitis, pain. Serious: Aplastic anemia, cholestatic jaundice syndrome, hepatic necrosis, toxic megacolon, angioedema.	Absorption: Oral 37% when fasting, 30% with food. Distribution: Protein binding absent. Distributed into bladder wall, kidneys, prostate, and seminal vesicles. Vd: 136.1 L. Metabolism: Converted to free acid fosfomycin. Excretion: Renal 38% (unchanged drug), fecal 18% (unchanged drug), dialyzable. Elimination half-life: 5.7 h.
Linezolid	Oxazolidinone	Inhibits bacterial protein synthesis by binding to 23S rRNA of the 50S subunit.	IV, PO	Common: Diarrhea, nausea/vomiting, headache. Serious: Hyponatremia, lactic acidosis, SIADH, *Clostridioides difficile* infection, myelosuppression, injury of liver, peripheral neuropathy, seizure, disorder of optic nerve, serotonin syndrome.	Absorption: IV 100%, oral 100%. Distribution: Protein binding 31%. Widely distributed. Very good CNS penetration but bacteriostatic. Vd: 50 L. Metabolism: Hepatic via oxidation. Excretion: Renal 30% (30% unchanged drug, 50% changed), non-renal 70% (Fecal 9% changed drug), dialyzable. Elimination half-life: 4.9 h.
Tedizolid	Oxazolidinone	Inhibits protein synthesis by binding to the 50S bacterial ribosomal subunit after conversion from prodrug.	IV, PO	Common: Phlebitis, diarrhea, nausea/vomiting, increased liver enzymes, dizziness, headache, infusion reaction. Serious: Tachycardia, *Clostridioides difficile* infection, colitis, neutropenia, peripheral nerve disease, disorder of optic nerve.	Absorption: IV 100%, Oral 91%. Distribution: Protein binding 70–90%. Widely distributed. Poor CNS penetration. Vd: 67–80 L. Metabolism: Tedizolid phosphate converted from prodrug to active drug, tedizolid. Excretion: Renal 18% (inactive conjugate), fecal 82% (inactive conjugate), non-dialyzable. Elimination half-life: 12 h.
Lefamulin	Pleuromutilin	Inhibits bacterial protein synthesis through interactions with the A- and P-sites of the 23S rRNA of the 50S subunit.	IV, PO	Common: Injection-site disorder, hypokalemia, diarrhea, nausea/vomiting, elevated liver enzymes, headache, insomnia. Serious: Prolonged QT interval, *Clostridioides difficile* infection.	Absorption: IV 100%, oral 25% (decreases slightly with food). Distribution: Protein binding 94.8–97.1%. Extensive in lung. Vd: IV, 86.1 L. Metabolism: Primarily CYP3A4. Excretion: IV = Renal 15.5% (9.6–14.1% unchanged), fecal 77.3% (4.2–9.1% unchanged). PO = renal 5.3% (% unchanged undetermined), fecal 88.5% (7.8–24.8% unchanged). Elimination half-life: 8 h.
Rifampin	Rifamycin	Blocks RNA transcription by binding to the beta subunit of DNA-dependent RNA polymerase, which inhibits bacterial RNA synthesis.	IV, PO	Common: None listed. Serious: Agranulocytosis, CID, thrombotic microangiopathy, hepatotoxicity, anaphylaxis, DRESS, nephrotoxicity, interstitial lung disease.	Absorption: IV 100%, PO well absorbed (decreases by 30% with food). Distribution: Protein binding 80%. Widely distributed. Highly lipophilic; crosses blood–brain barrier well. Vd: 0.66 L/kg (300 mg); 0.64 L/kg (600 mg). Metabolism: Hepatic metabolism to deacetylated metabolite. Undergoes enterohepatic recirculation. Potent 3A4 inducer. Excretion: Renal ≤ 30%, fecal 60–65% (unchanged drug) Elimination half-life: 2–5 h.
Quinupristin/dalfopristin	Streptogramin	Interrupts protein synthesis, as each agent binds at a different site of the bacterial 50S ribosomal subunit.	IV	Common: Injection-site disorders, rash, diarrhea, nausea/vomiting, thrombophlebitis, conjugated hyperbilirubinemia, hyperbilirubinemia, arthralgia, myalgia. Serious: Generalized myasthenia, hepatitis, GI hemorrhage, cerebral hemorrhage, myocardial infarction.	Absorption: IV 100% Distribution: Protein binding 50–56%. Extensive, including tissue, blister fluid, and CNS penetration. Vd: Quinupristin, 0.45 L/kg; dalfopristin, 0.24 L/kg. Metabolism: Quinupristin conjugated to active metabolites in liver; dalfopristin hydrolyzed to active metabolites. Excretion: Quinupristin = renal 15%, fecal 75–77%. Dalfopristin = renal 19%. non-dialyzable. Elimination half-life: Quinupristin, 0.85 h. Dalfopristin, 0.7 h.
Trimethoprim/sulfamethoxazole	Sulfonamide derivative	TMP acts synergistically with SMX by interfering sequentially in the folic acid production pathway.	IV, PO	Common: Rash, urticaria, loss of appetite, nausea/vomiting. Serious: Cardiogenic shock, prolonged QT interval, torsades de pointes, ventricular tachycardia, acute renal failure, rhabdomyolysis, DRESS, aplastic anemia, thrombocytopenia, cutaneous drug reactions (erythema multiforme, SJS, TEN, Sweet’s syndrome).	Absorption: IV 100%, PO 90–100%. Distribution: Protein binding; SMX 70%, TMP 44–62%. Widely distributed. Crosses blood–brain barrier. Vd: SMX 360 mL/kg, TMP 2 L/kg. Metabolism: Hepatic metabolism to multiple metabolites. Free forms of both SMX and TMP are therapeutically active. Excretion: Renal = SMX 10–30%, TMP 50–70%, dialyzable. Elimination half-life: SMX 9–13 h, TMP 8–11 h.
Doxycycline	Tetracycline	Inhibits protein synthesis by binding to the 30S ribosomal subunit.	IV, PO	Common: Rash, diarrhea, loss of appetite, nausea/vomiting, sensitive dentin/gums, myalgia, bacterial vaginosis. Serious: DRESS, erythema multiforme, photosensitivity, SJS, TEN, *Clostridium difficile* diarrhea, superinfection, pseudotumor cerebri, anaphylaxis, arrest of bone development and/or growth.	Absorption: IV 100%, PO 100%. Distribution: Protein binding > 90%. Widely distributed into tissues and fluids. Vd: 1.36 L/kg. Metabolism: Intestinal inactivation by chelate formation. Excretion: Renal 23–40%, fecal 30%, non-dialyzable. Elimination half-life: 18–22 h.
Eravacycline	Tetracycline	Inhibits protein synthesis by binding to the 30S ribosomal subunit.	IV	Common: Infusion reaction, nausea/vomiting. Serious: Photosensitivity, acidosis, hyperphosphatemia, *Clostridium difficile* diarrhea, necrosis of pancreas, acute pancreatitis, staining of tooth, abnormal liver function, hypersensitivity reaction, pseudotumor cerebri, azotemia, BUN increase.	Absorption: IV 100%. Distribution: Protein binding 79–90%. Extensive into tissues. Vd: 321 L. Metabolism: CYP3A4 metabolism and FMO-mediated oxidation. Excretion: Renal 34% (20% unchanged), fecal 47% (17% unchanged). Elimination half-life: 20 h.
Minocycline	Tetracycline	Inhibits protein synthesis by binding to the 30S and possibly the 50S ribosomal subunit(s).	IV, PO	Common: Pruritus, dizziness, headache, fatigue. Serious: Fixed drug eruption, SJS, acidosis, hyperphosphatemia, *Clostridioides difficile* infection, enamel hypoplasia, tooth discoloration, autoimmune hepatitis, acute hepatic failure, hepatitis, anaphylaxis, DRESS, systemic lupus erythematosus, pseudotumor cerebri, azotemia, BUN elevation, serum sickness.	Absorption: IV 100%, PO well absorbed. Distribution: Protein binding 55–96%. Widely distributed. Poor CNS penetration Vd: 0.14–0.7 L/kg. Metabolism: Hepatic to inactive metabolites. Excretion: Renal 4–19%, fecal 20–34%, non-dialyzable. Elimination half-life: IV 15–23 h, PO 16 h.
Omadacycline	Tetracycline	Inhibits protein synthesis by binding to the 30S ribosomal subunit.	IV, PO	Common: Infusion reaction, constipation, nausea/vomiting, ALT/SGPT elevation, AST elevation, GGT elevation, headache. Serious: Photosensitivity, acidosis, hyperphosphatemia, *Clostridium difficile* diarrhea, acute pancreatitis, staining of tooth, abnormal liver function, hypersensitivity reaction, arrest of bone development and/or growth, azotemia, BUN elevation, death.	Absorption: IV 100%, PO 34.5%. Distribution: Protein binding 20%. Tissue concentrations exceed plasma concentrations in alveolar cells and epithelial lining fluid. Vd: IV 190 L, PO 794 L. Metabolism: Not metabolized. Excretion: IV = renal 27%. PO = renal 14.4%, fecal 77.5–80%. Dialyzable. Elimination half-life: IV 16 h, PO 13.45–16.83 h.

Footnote: PO, oral (per os); IV, intravenous; IM, intramuscular; Vd, volume of distribution.

**Table 2:** Antimicrobials to treat MRSA infections: Adult and pediatric dosing [41,51,52,53,54,55].

Medication	Adult Dose (MRSA Indications Only)	Pediatric Dose (MRSA Indications Only)	Neonatal Dose (MRSA Indications Only)
Ceftaroline	SSTI, community-acquired pneumonia (alternative agent, combination therapy), hospital-acquired or ventilator-associated pneumonia (alternative agent, off-label): 600 mg IV every 12 h bacteremia (alternative agent, off-label), endocarditis (salvage therapy, off-label): 600 mg IV every 8 h as part of an appropriate combination regimen Renal dose adjustment required.	SSTI (standard dose, high dose recommended for MIC 2 or 4 mg/L), community-acquired pneumonia (≥2 months): ≥2 months to <2 years: 8 mg/kg IV every 8 h, high dose 10 mg/kg IV every 8 h ≥2 years to ≤33 kg: 12 mg/kg IV every 8 h, high dose 12 mg/kg IV every 8 h (max 600 mg/dose) ≥2 years and >33 kg: 400 mg IV every 8 h OR 600 mg IV every 12 h, high dose 12 mg/kg IV every 8 h (max 600 mg/dose) Bacteremia (limited data): 2 months to <6 months: 10 mg/kg IV every 8 h ≥6 months, children, adolescents: 15 mg/kg IV every 8 h (max 600 mg/dose) Osteomyelitis (limited data): 1 month to <18 years: 15 mg/kg IV every 8 h (max 600 mg/dose) Renal dose adjustment required.	SSTI: 34 weeks and 12 days postnatal to <2 months: 6 mg/kg IV every 8 h General dosing (limited data): ≤2 kg and ≤7 days postnatal: 6 mg/kg IV every 12 h ≤2 kg and 8–60 days postnatal: 6 mg/kg IV every 8 h >2 kg and ≤7 days postnatal, >2 kg and 8–60 days postnatal: 6 mg/kg IV every 8 h Renal dose adjustment required.
Ceftobiprole	Bacteremia: 667 mg ceftobiprole medocaril IV every 6 h × 8 days, followed by 667 mg IV every 8 h thereafter SSTI, community-acquired pneumonia (combination therapy), hospital-acquired pneumonia (alternative agent, off-label): 667 mg ceftobiprole medocaril IV every 8 h Renal dose adjustment required.	Community-acquired pneumonia: ≥3 months to <12 years: 20 mg/kg ceftobiprole medocaril IV every 8 h (max 667 mg/dose) ≥12 years to <18 years: 13.3 mg/kg ceftobiprole medocaril IV every 8 h (max 667 mg/dose) Renal dose adjustment required.	Dosing guidelines not available
Daptomycin	Bacteremia, CSF shunt infection (alternative agent, off-label), intracranial abscess, spinal epidural abscess (alternative agent, off-label), meningitis, osteomyelitis, prosthetic joint infection (alternative agent, off-label): 6–10 mg/kg IV once daily (guideline dosage) Diabetic foot, SSTI (alternative agent), spontaneous bacterial peritonitis, febrile neutropenia: 4–6 mg/kg IV once daily endocarditis (alternative agent): 8–12 mg/kg IV once daily Septic arthritis (alternative agent, off-label): 6–10 mg/kg IV once daily Renal dose adjustment required. Do not use for pneumonia treatment.	Osteomyelitis, bacterial arthritis, bacteremia (standard and high dose): 1 to 6 years: 12 mg/kg IV daily 7 to 11 years: 9 mg/kg IV daily, high dose 10–12 mg/kg IV daily 12 to 17 years: 7 mg/kg IV daily, high dose 8–10 mg/kg IV daily SSTI: 1 to <2 years: 10 mg/kg IV daily 2 to 6 years: 9 mg/kg IV daily 7 to 11 years: 7 mg/kg IV daily 12 to 17 years: 5 mg/kg IV daily Endocarditis: <6 years: 10–12 mg/kg IV daily ≥6 years: 8–12 mg/kg IV daily Renal dose adjustment required. Do not use for pneumonia treatment.	Dosing guidelines not available
Delafloxacin	Community-acquired pneumonia (alternative agent), SSTI: Oral: 450 mg every 12 h; IV: 300 mg every 12 h Renal dose adjustment required.	Dosing guidelines not available	Dosing guidelines not available
Dalbavancin	SSTI (alternative agent): 1500 mg IV once OR 1000 mg IV once, followed by 500 mg IV once 1 week later Osteomyelitis (alternative agent, off-label): 1500 mg IV weekly × 2 weeks Bacteremia (alternative agent, off-label): 1000–1500 mg IV once OR 1000 mg IV once, followed by 500 mg IV once 1 week later OR 1500 mg IV weekly × 2 weeks Endocarditis (alternative agent, off-label): 1000 mg IV once, followed by 500 mg IV weekly OR 1500 mg IV once, followed by 1000 mg IV every other week Renal dose adjustment required.	SSTI: Infants and children < 6 years: 22.5 mg/kg IV once (max 1500 mg/dose) ≥6 years and adolescents: 18 mg/kg IV once (max 1500 mg/dose) Renal dose adjustment may be required.	SSTI: 22.5 mg/kg IV once (max 1500 mg/dose) Insufficient data on renal dose adjustment.
Oritavancin	SSTI: 1200 mg IV once Endocarditis (alternative agent, off-label): 1200 mg IV weekly	Dosing guidelines not available	Dosing guidelines not available
Teicoplanin (not available in US)	Gram-positive infections (bacteremia, SSTI, UTI, community-acquired pneumonia, hospital-acquired pneumonia, bone/joint infections, and endocarditis): 6–12 mg/kg IV every 12 h for 3–5 doses, followed by 6–12 mg/kg IV/IM daily to achieve target concentration	Gram-positive infections (bacteremia, SSTI, UTI, community-acquired pneumonia, hospital-acquired pneumonia, bone/joint infections, and Endocarditis): Infant < 2 months: 16 mg/kg IV once, followed by 8 mg/kg IV daily ≥2 months to ≤12 years: 10 mg/kg IV every 12 h for 3 doses followed by 6–10 mg/kg IV daily Adolescents: 6–12 mg/kg IV every 12 h for 3–5 doses, followed by 6–12 mg/kg IV/IM daily to achieve target concentration	Dosing guidelines not available
Telavancin	Bacteremia (alternative agent, off-label), hospital-acquired or ventilator-associated pneumonia (alternative agent), SSTI (alternative agent): 10 mg/kg IV daily Renal dose adjustment required.	Dosing guidelines not available	Dosing guidelines not available
Vancomycin	Bacteremia, CSF shunt infection (off-label), diabetic foot infection (off-label), endocarditis (alternative agent), intra-abdominal infection (off-label), intracranial abscess. Epidural abscess (off-label), meningitis (off-label), osteomyelitis, pneumonia, prosthetic joint infection (off-label), sepsis/septic shock, septic arthritis, SSTI, toxic shock syndrome: 10–20 mg/kg IV every 8 to 48 h. May consider bolus of 20–35 mg/kg in seriously ill patients. Requires AUC monitoring to guide proper dose. Generally, AUC values around 400 are considered appropriate for most indications. AUC values closer to 600 may be considered for meningitis or endocarditis. Renal dose adjustment required.	Endocarditis: Children to adolescents: 40 mg/kg/day IV divided every 8–12 h Meningitis: Infants to adolescents: limited data, 15 mg/kg/dose IV every 6 h Osteoarticular infection, pneumonia: Infants > 3 months to adolescents: 40–60 mg/kg/day divided every 6–8 h. SSTI: Infant 1 month to <3 months: 10 mg/kg IV every 6 h Infant ≥ 3 months to <12 years: 60–80 mg/kg/day IV divided every 6 h ≥12 years to adolescents: 60–70 mg/kg/day IV divided every 6- 8 h Requires AUC monitoring to guide proper dose. Generally, AUC values around 400 are considered appropriate for most indications. AUC values closer to 600 may be considered for meningitis or endocarditis. If trough levels are utilized, values around 5–15 mg/L are recommended based on indication. Renal dose adjustment required.	Serious MRSA infections: 10–20 mg/kg/dose IV every 8–48 h depending on postmenstrual age, weight, and serum creatinine. Requires AUC monitoring to guide proper dose. Generally, AUC values around 400 are considered appropriate for most indications. If trough levels are utilized, values around 5–15 mg/L are generally recommended based on indication. Renal dose adjustment required.
Tigecycline	Community-acquired pneumonia, diverticulitis, febrile neutropenia, SSTI, intra-abdominal infection: 100 mg IV once, followed by 50 mg IV every 12 h	General dosing, bacterial infection: Limited data available. Reserve for situations when no effective alternative is available. Infants to <8 years: Not recommended due to adverse effects; however, use described when no alternatives available. 1.5–2 mg/kg IV loading dose once, followed by 1–2 mg/kg/dose IV every 12 h (max 50 mg/dose) ≥8 years to adolescents: 1.5–2 mg/kg IV loading dose once, followed by 1.2–2 mg/kg/dose IV every 12 h (max 50 mg/dose)	Dosing guidelines not available
Clindamycin	Diabetic foot infection, SSTI: 600–900 mg IV every 8 h, 300 mg PO four times daily or 450 mg PO three times daily Septic arthritis, pneumonia (alternative therapy): 600 mg IV/PO every 8 h Osteomyelitis: 600–900 mg IV/PO every 8 h Sepsis: 600–2700 mg/day IV divided into 2–4 equal doses Toxic shock syndrome, toxin production suppression: 900 mg IV every 8 h	SSTI: 25–40 mg/kg/day IV divided every 8 h, 20–40 mg/kg/day PO divided every 6–8 h depending on infection. Community-acquired pneumonia: Infants > 3 months to adolescents: 40 mg/kg/day IV divided every 6–8 h, 30–40 mg/kg/day PO divided every 6–8 h Osteoarticular infection: Infants to adolescents: 30–40 mg/kg/day IV/PO divided every 6–8 h (max IV 900 mg/dose, PO 600 mg/dose) Sepsis: 1 month to 16 years: 20–40 mg/kg/day IV divided into 3- 4 equal doses Toxic shock syndrome, toxin production suppression: Infants to adolescents: 40 mg/kg/day in divided doses every 6–8 h (max 900 mg/dose)	General dosing, SSTI: Age directed IV/PO ≤32 weeks: 5 mg/kg/dose every 8 h >32 weeks to 40 weeks: 7 mg/kg/dose every 8 h >40 weeks: 9 mg/kg/dose every 8 h General dosing, SSTI: Weight-directed IV/IM/PO ≤2 kg and ≤28 days: 5 mg/kg/dose every 8 h ≤2 kg and 29–60 days: 10 mg/kg/dose every 8 h >2 kg and ≤7 days: 7 mg/kg/dose every 8 h >2 kg and 8–28 days: 9 mg/kg/dose every 8 h >2 kg and 29–60 days: 10 mg/kg/dose every 8 h
Fosfomycin (IV not available in United States)	Bacteremia, bone and joint infection, endocarditis, intra-abdominal infection, hospital-acquired or ventilator-associated pneumonia, SSTI: 12–24 g/day IV divided into 2–3 doses Meningitis: 16–24 g IV divided into 2–3 doses Renal dose adjustment required.	Bone and joint infection, endocarditis, intra-abdominal infection, hospital-acquired or ventilator-associated pneumonia, meningitis, SSTI: <10 kg: 200–300 mg/kg/day IV in 3 divided doses 10–40 kg: 200–400 mg/kg/day IV in 3–4 divided doses >40 kg: 12–24 g/day IV divided into 2–3 doses Renal dose adjustment required.	Dosing guidelines not available
Linezolid	Bacteremia (alternative agent, off-label), CNS infection (alternative agent, off-label), cystic fibrosis pulmonary exacerbation (off-label), diabetic foot infection, endocarditis (off-label), intracranial abscess (alternative agent, off-label), meningitis (off-label), osteomyelitis (alternative agent, off-label), pneumonia, prosthetic joint infection (alternative agent, off-label), septic arthritis (alternative agent, off-label), SSTI (alternative agent), toxic shock syndrome (off-label): 600 mg IV/PO every 12 h	Bacteremia, meningitis (alternative agent), osteoarticular infection, pneumonia, SSTI (complicated): Infants to <12 years: 10 mg/kg/dose IV/PO every 8 h ≥12 years: 10 mg/kg/dose IV/PO every 12 h (max 600 mg/dose) SSTI (uncomplicated): Infants to <5 years: 10 mg/kg/dose PO every 8 h 5 to 11 years: 10 mg/kg/dose PO every 12 h (max 600 mg/dose) ≥12 years: 600 mg PO every 12 h	General dosing, SSTI, pneumonia: Gestational age < 34 weeks and chronological age < 7 days: 10 mg/kg/dose IV/PO every 12 h Gestational age < 34 weeks and chronological age > 7 days: 10 mg/kg/dose IV/PO every 8 h Gestational age ≥ 34 weeks: 10 mg/kg/dose IV/PO every 12 h
Tedizolid	SSTI: 200 mg IV/PO daily	SSTI: Children to adolescent ≥ 35 kg: 200 mg PO daily 1 to <2 kg: 3 mg/kg/dose IV every 12 h 2 to <3 kg: 6 mg/kg/dose IV every 12 h 3 to <6 kg: 12 mg/kg/dose IV every 12 h 6 to <10 kg: 20 mg/kg/dose IV every 12 h 10 to <14 kg: 30 mg/kg/dose IV every 12 h 14 to <20 kg: 40 mg/kg/dose IV every 12 h 20 to <35 kg: 60 mg/kg/dose IV every 12 h ≥35 kg: 200 mg IV daily	SSTI: ≥26 weeks gestational age 1 to <2 kg: 3 mg/kg/dose IV every 12 h 2 to <3 kg: 6 mg/kg/dose IV every 12 h 3 to <6 kg: 12 mg/kg/dose IV every 12 h 6 to <10 kg: 20 mg/kg/dose IV every 12 h
Lefamulin (not available in US)	Community-acquired pneumonia: 150 mg IV every 12 h or 600 mg PO every 12 h	Dosing guidelines not available	Dosing guidelines not available
Rifampin	Meningitis (adjunctive agent, off-label): 600 mg IV daily Endocarditis (adjunctive agent, off-label): 300 mg IV/PO every 8 h Osteomyelitis (adjunctive agent, off-label): 450–600 mg PO daily Prosthetic joint infection (adjunctive agent, off-label): 300–450 mg PO twice daily	Endocarditis (adjunctive agent, off-label): Infant to adolescents: 15–20 mg/kg/day IV/PO divided every 8–12 h (max 900 mg/day) Meningitis (adjunctive agent, off-label): >28 days: 10–20 mg/kg/day IV divided every 12–24 h	Meningitis (adjunctive agent, off-label): 8 to 28 days: 10–20 mg/kg/day IV divided every 12 h Staphylococcal infections, persistent (adjunctive agent): 10–20 mg/kg PO every 24 h or 5–10 mg/kg/dose IV every 12 h
Quinupristin/dalfopristin (no longer marketed)	Bacteremia (alternative agent, off-label), healthcare-associated CNS infection (alternative agent, off-label): 7.5 mg/kg IV every 8 h SSTI: 7.5 mg/kg IV every 12 h	MRSA (salvage therapy, limited data): Infant to adolescents: 7.5 mg/kg IV every 8 h SSTI (limited data on infants and children < 12 years): Infant to adolescents: 7.5 mg/kg IV every 12 h	General dosing: 7.5 mg/kg IV every 12 h
Trimethoprim/sulfamethoxazole	Diabetic foot infection (off-label), SSTI: 1–2 double strength (DS) tablets PO twice daily Endocarditis (alternative agent, off-label): 2 DS tablets PO twice daily Intra-abdominal infection (alternative agent, off-label), mastitis (alternative agent, off-label), prosthetic joint infection (off-label): 1 DS tablet twice daily Intracranial abscess (alternative age, off-label), meningitis (alternative therapy): 5 mg/kg/dose (TMP component) IV every 8–12 h Osteomyelitis (alternative agent, off-label), septic arthritis (off-label): 1–2 DS tablets PO twice daily or 4 mg/kg/dose (TMP component) IV every 12 h Renal dose adjustment required.	Meningitis (alternative agent): ≥2 months to adolescents: 10–20 mg/kg/day IV divided every 6- 12 h Osteoarticular infection: ≥2 months to adolescents: 6–20 mg TMP/kg/day PO divided every 6–12 h (max 160 mg TMP/dose) SSTI: ≥2 months to adolescents: 8–12 mg TMP/kg/day IV/PO divided every 12 h (max 320 mg TMP/dose) Renal dose adjustment required.	Dosing guidelines not available
Doxycycline	Diabetic foot infection, endocarditis (alternative agent, off-label), prosthetic joint infection (off-label), septic arthritis (off-label), SSTI: 100 mg PO twice daily Community-acquired pneumonia (alternative agent): 100 mg IV/PO twice daily	Community-acquired pneumonia: ≥8 years to adolescents: 1–2 mg/kg/dose PO twice daily SSTI: ≥8 years to adolescents and ≤45 kg: 2 mg/kg/dose PO twice daily ≥8 years to adolescents and >45 kg: 100 mg PO twice daily	Dosing guidelines not available
Eravacycline	Intra-abdominal infection (alternative agent): 1 mg/kg IV every 12 h or 1.5 mg/kg IV every 24 h	Dosing guidelines not available	Dosing guidelines not available
Minocycline	Prosthetic joint infection (alternative agent, off-label): 100 mg PO twice daily Septic arthritis (off-label), SSTI: 200 mg PO once, followed by 100 mg PO twice daily	SSTI: >8 years and adolescents: 4 mg/kg PO once then 2 mg/kg/dose PO every 12 h	Dosing guidelines not available
Omadacycline	Community-acquired pneumonia (alternative agent): 200 mg IV once or 100 mg IV twice daily on day 1, followed by 100 mg daily, 300 mg PO twice daily on day 1, followed by 300 mg PO daily SSTI: 200 mg IV once or 100 mg IV twice daily on day 1, followed by 100 mg daily, 450 mg PO daily on day 1, and 2 followed by 300 mg PO daily	Dosing guidelines not available	Dosing guidelines not available

Footnote: PO, oral (per os); IV, intravenous; SSTI, skin and soft tissue infections; DS, double strength; TMP, trimethoprim.

## Data Availability

No new data were generated, created or analyzed in this study. Data sharing is not applicable to this article.
